# Formulation and In Vitro Evaluation of Mucoadhesive Sustained Release Gels of Phytoestrogen Diarylheptanoids from *Curcuma comosa* for Vaginal Delivery

**DOI:** 10.3390/pharmaceutics15010264

**Published:** 2023-01-12

**Authors:** Ekapol Limpongsa, Peera Tabboon, Sarunya Tuntiyasawasdikul, Bungorn Sripanidkulchai, Thaned Pongjanyakul, Napaphak Jaipakdee

**Affiliations:** 1 College of Pharmacy, Rangsit University, Pathum Thani 12000, Thailand; 2 Faculty of Pharmaceutical Sciences, Khon Kaen University, Khon Kaen 40002, Thailand; 3 Center for Research and Development of Herbal Health Products, Khon Kaen University, Khon Kaen 40002, Thailand

**Keywords:** *Curcuma comosa*, phytoestrogens, mucoadhesive gels, polyacrylic acid, vaginal gels, zero-order release

## Abstract

Diarylheptanoids (DAs) characterized by a 1,7-diphenylheptane structural skeleton are considered a novel class of phytoestrogens. The DAs available in *Curcuma comosa* Roxb. (*C. comosa*) extract demonstrated significant estrogenic activities both in vitro and in vivo. This study aimed to develop and comprehensively evaluate a mucoadhesive vaginal gel for the sustained release of DAs. Different mucoadhesive polymers as gelling agents were investigated. *C. comosa* ethanolic crude extract was used as a source of DAs. All *C. comosa* gels were light brown homogeneous with pH within 4.4–4.6. Their flow behaviors were pseudoplastic with a flow behavior index of 0.18–0.38. The viscosity at a low shear rate varied from 6.2 to 335.4 Pa·s. Their mechanical and extrudability properties were associated well with rheological properties. Polycarbophil (PCP):hydroxypropyl methylcellulose (HPMC) blends had a higher mucoadhesiveness to porcine vaginal mucosa than those of PCP-based or HPMC-based gels. All *C. comosa* gels exhibited a sustained, zero-order DA release pattern over 72 h. Korsmeyer and Peppas equation fitting indicated a non-Fickian, case II transport release mechanism. *C. comosa* gels had good physical and chemical stability under low-temperature storage for up to 12 months. PCP:HPMC-based mucoadhesive gels could be a proper delivery system for vaginal administration of DAs.

## 1. Introduction

There is a growing body of evidence approving the use of phytoestrogens for menopausal-related symptom alleviation. Phytoestrogens are plant-derived compounds that are structurally or pharmacologically actively analogous to mammalian estrogens and, therefore, able to permit binding to estrogen receptors in animals and humans [1,2]. The beneficial efficacy and safety of topical phytoestrogen-containing vaginal formulations—*Glycine Max* isoflavone gels and *Pueraria mirifica* gels—on the restoration of the symptoms associated with the genitourinary syndrome of menopause have been demonstrated [2,3,4,5,6].

**Figure 1:**
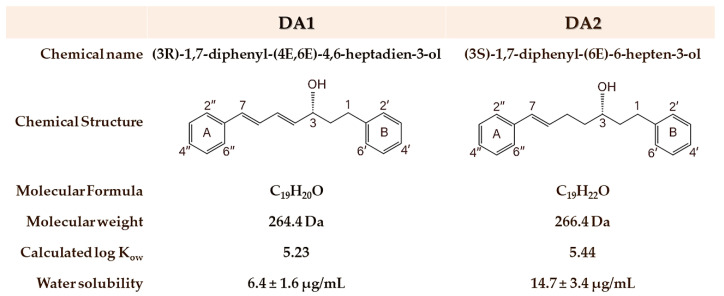
Structures and physicochemical properties of DA1 and DA2 presented in *C. comosa* extract. Log *K*_ow_ refers to a log octanol/water partition coefficient calculated via KOWWIN v1.68 estimate. Water solubility at 37 °C is modified from Jaipakdee et al. [14].

Diarylheptanoids (DAs), a novel class of phytoestrogens characterized by a 1,7-diphenylheptane structural skeleton, are acknowledged as promising therapeutic compounds due to their miscellaneous pharmaceutical activities—estrogenic, anti-inflammatory, antitumor, antioxidant, as well as neuroprotective activities, etc. [7]. DAs obtained from the rhizomes of *Curcuma comosa* Roxb. *(C. comosa)* have been described to manifest various estrogenic actions mediated through estrogen receptors. Several investigations indicated that *C. comosa* extract and its major active compounds—(3R)-1,7-diphenyl-(4E,6E)-4,6-heptadien-3-ol (DA1) and (3S)-1,7-diphenyl-(6E)-6-hepten-3-ol (DA2) (Figure 1)—demonstrated significant estrogenic activities both in vitro and in vivo [8,9,10,11,12]. DA1 was identified as the most potent phytoestrogenic compound with a relative efficacy of approximately 4% of 17β-estradiol. The existence of a keto group at C3 and a lack of hydroxyl group in ring B of DAs were claimed to be responsible for their estrogenic activity [8,9]. The considerable quantity of DAs available in its extract suggests *C. comosa* as an interesting phytoestrogen source [12].

*C. comosa* taxonomically is a member of the *Curcuma* genus of the *Zingiberaceae* family and is principally grown in the tropics of Asia. Traditionally, *C. comosa* has been utilized to alleviate gynecological disorders as well as menopausal symptoms. At present, it is widely utilized as a dietary supplement product, in the form of oral capsules and liquids, for health promotion as well as hormone replacement therapy in peri- and postmenopausal women in Thailand [7]. A small number of drug delivery systems of *C. comosa* have been developed including peroral nanoemulsions [13], liquisolid peroral tablets [14], transdermal films [15], transfersomal gels [16], and electrospun gelatin nanofibers [17]. Recently, Tunpanich et al. [18] investigated the mucoadhesive vaginal tablets of *C. comosa* for local delivery of DAs using polycarbophil (PCP), hydroxypropylmethylcellulose (HPMC), or the mixture of PCP and HPMC as mucoadhesive and sustained release polymers. The established *C. comosa* vaginal tablets exhibited a sustained release of DAs for up to 72 h with the cumulative percent release at 72 h ranging from 17 to 83% and 34 to 94% for DA1 and DA2, respectively.

The vaginal route of administration is a valuable route for local drug delivery. Local administration of estrogenic compounds to the vaginal cavity tissues has been favored in the treatment of the symptoms associated with the genitourinary syndrome of menopause. To accomplish a desirable restorative effect, vaginal delivery systems must contain a capacity to spread onto and develop intimate contact with the vaginal mucosa [19,20,21,22]. It is known that the conventional semisolid dosage forms—gels, creams, etc.—contribute a poor adhesion and thus short retention in the vaginal tract due to the presence of moisture, lubricative mucus, and shear forces. A mucoadhesive sustained-release gel may be considered an optimal system design to enhance both therapeutic efficacy and patient adherence. Gels are well-accepted dosage forms for vaginal applications of numerous active pharmaceutical ingredients [23]. Mucoadhesive gel possesses a high ability to adhere to mucosal surfaces lengthening the retention time at the application site and reducing the number of applications required [21,24,25]. The three-dimensional networks of mucoadhesive polymer and/or gelling agents possess the ability to provide sustained release behavior of the loaded active ingredients [22]. Additionally, the hydrogel-based formulation, due to its high-water content, is able to function as a moisturizer providing hydration and relieving vaginal dryness symptoms experienced especially during menopause [23]. Mucoadhesive vaginal gels offer particular properties that can be utilized as a drug delivery system for local drug administration of phytoestrogens to achieve desirable therapeutic effects.

A critical component of mucoadhesive gels is the mucoadhesive polymer that possesses the capability to physically and chemically interact with mucin glycoproteins in the mucus layer located on the mucosal epithelium. The physical interaction is caused by the interpenetration, entanglement, and mechanical interlocking between the chains of mucoadhesive polymer and mucin glycoproteins, whereas chemical interaction is the association of mucoadhesive polymer and mucins, depending on the chemical structure of the polymers, through van der Waals, hydrophobic, hydrogen bonds, and/or electrostatic interactions. The formation of hydrogen bonds among the polymers and mucins imparts a major role in strong mucoadhesion. With this regard, the presence of a large number of hydrogen bond-forming moieties—carboxyl, hydroxyl, amine, and sulfate groups—is considered the most significant feature of potentially mucoadhesive polymers [21,26,27].

Mucoadhesive polymers, either water-soluble or water-insoluble, are swellable hydrophilic polymers. Numerous mucoadhesive polymers that are capable of forming hydrogels have been investigated in vaginal delivery. Amongst these mucoadhesives, polyacrylic acid (PAA)—carbomer and PCP—are the most commonly investigated mucoadhesive polymers for vaginal preparations [20,21,23,27]. Numerous preparations fabricated from these PAA have successfully been commercialized, e.g., Replens^®^ gel (vaginal moisturizer) and Crinone^®^ gel (progesterone vaginal gel). Carbomers are high molecular weight polymers of acrylic acid highly cross-linked with allyl pentaerythritol, whereas PCP is of high molecular weight of acrylic acid lightly cross-linked with divinyl glycol [28]. Both carbomers and PCP are anionic polymers due to the considerable number of carboxyl groups contained in their molecules. PAA are water-insoluble but possess excellent water-absorbing and excessive swelling capacities, especially upon neutral pH conditions. The excessive swelling as well as hydrogen bond forming ability render excellent mucoadhesive properties of PAA. Additionally, the high swelling property of PAA significantly encloses their capacity as a sustained release carrier [26,27]. Pedersen et al. [29] developed a gel based on 1–2% of a PAA blend, carbomer 974P:PCP (1:2), for a poorly water-soluble salicylidene acylhydrazide delivery. The fabricated gels could slow the release of the associated drug by 20-fold as compared to that from the DMSO/buffer solution. Nevertheless, the rheological, mechanical, and mucoadhesive characteristics of PAA-based gels depend upon the environmental pH. Combinations with other mucoadhesive polymers such as cellulose ether derivative, e.g., HPMC, could limit the pH sensitivity of the system [30]. HPMC is a water-soluble cellulose derivative with numerous pharmaceutical applications including sustained release capability in intravaginal drug delivery. PAA and/or cellulose derivatives are one of the most commonly used polymers in commercial vaginal products [21,31]. The vaginal gels prepared with a combination of carbomer 974P and hydroxyethylcellulose were successfully used to deliver tenofovir–nonnucleoside reverse-transcriptase inhibitor UC781. The ratio of carbomer 974P and hydroxyethylcellulose influenced the release rate of UC781. The release of associated UC781 from the carbomer 974P:hydroxyethylcellulose gels could be sustained due to its low water solubility characteristics and the high viscous gel network [32].

The objective of this study was to develop and comprehensively evaluate a mucoadhesive vaginal gel for the sustained release of DAs. *C. comosa* ethanolic crude extract was used as a source of phytoestrogen DAs. For this purpose, different types of mucoadhesive polymers—PCP, C980, HPMC K15M, and HPMC K100M—were investigated. The rheological profile, mechanical, extrudability, and mucoadhesiveness of the prepared gels were assessed. Release behaviors of DA1 and DA2 were determined and kinetically evaluated. The accelerated and long-term stability of the selected *C. comosa* gels was also evaluated.

## 2. Materials and Methods

### 2.1. Materials

*Curcuma comosa* (*C. comosa*) dry rhizomes, harvested from Kampaengsaen district, Nakhon Pathom, Thailand, were supplied by Professor Pawinee Piyachaturawat (Mahidol University, Bangkok, Thailand). Standard DA1 and DA2 were obtained from Professor Apichart Suksamrarn (Ramkhamhaeng University, Bangkok, Thailand). Hydroxypropyl methylcellulose K15M (HPMC K15M, Benecel^TM^, Ashland, Worcestershire, England) and hydroxypropyl methylcellulose K100M (HPMC K100M, Benecel^TM^, Ashland, Worcestershire, England) were obtained as a gift from Rama Production, Co., Ltd. (Bangkok, Thailand). Polycarbophil (PCP, Noveon^®^ AA-1, Lubrizol Corporation, Wickliffe, OH, USA) was provided by NamSiang Co. Ltd. (Bangkok, Thailand). Carbomer (Carbopol^®^ 980, C980) was purchased from Wanrat (Namsiang) (Bangkok, Thailand). Methylparaben was purchased from Sigma-Aldrich (St. Louis, MO, USA). Glycerol and polyethylene glycol 400 (PEG 400) were purchased from Merck KGaA (Darmstadt, Germany). Ethyl alcohol (95%) was purchased from the Liquor Distillery Organization (Chachoengsao, Thailand). Polysorbate 80 (polyoxyethylene sorbitan monooleate) was purchased from AppliChem GmbH (Darmstadt, Germany). Sodium hydroxide, as well as high-performance liquid chromatography (HPLC) grade ethanol, acetonitrile, and methanol, were purchased from RCI Labscan (Bangkok, Thailand). All ingredients were used as received.

### 2.2. Preparation of C. comosa Extract

The ethanolic crude extract of *C. comosa* rhizome was produced via the maceration method [18] followed by the decolorization and defatting treatments. Briefly, the pulverized *C. comosa* was soaked in ethyl alcohol (95%) for 7 days with periodic stirring (2–3 times daily) before passing through Whatman^®^ filter paper. The filtrate was collected and evaporated using a rotary vacuum evaporator (SB-1000, Eyela^®^, Tokyo, Japan) to a constant weight. For the decolorization process, the obtained red-brown oleoresin-like extract was redissolved in absolute ethanol (1:3) and stirred with granular activated carbon for 5 min by using a magnetic stirrer (Ahn^®^, myLab SM-10, Nordhausen, Germany) before being filtrated with Whatman filter paper (No.1). The waxy material of the obtained filtrate was removed via winterization [33] by cooling the obtained filtrate in the dry ice-ethanol cooling bath for 6 h. Then, the supernatant was filtered through Whatman filter paper (No.1) and dried at 40 °C by using a rotary evaporator. The 6.8% yield, dark brown liquid extract containing 16.7 ± 0.2% of DA1 and 16.3 ± 0.1% of DA2 was obtained. The obtained *C. comosa* extract was stored at −40 °C until utilized.

### 2.3. Preparation of C. comosa Gels

Different *C. comosa* gels based on different mucoadhesive polymers as a gelling agent, as presented in Table 1, were prepared. Briefly, *C. comosa* extract mixture was firstly prepared by blending *C. comosa* extract with polysorbate 80 and then with the solution of methylparaben in glycerol. For HPMC-based gels, the required amount of HPMC was firstly dispersed in the 90 °C deionized water. This dispersion was gently stirred (model HS 10-2, Torrey Pines Scientific, Carlsbad, CA, USA) until its temperature was approximately 50 °C. This warmed HPMC aqueous dispersion was then added to the *C. comosa* extract mixture under continuous stirring. The stirring was performed to let the HPMC swell during cooling down, and a homogeneous gel was obtained. Sodium hydroxide solution (4%) was then added to adjust the pH of the mixture to 4.5 ± 0.1.

For PCP-based and C980-based gels, the polymer was dispersed and swollen in deionized water under mechanical stirring (model HS 10-2, Torrey Pines Scientific, Carlsbad, CA, USA). The resulting polymer dispersion was gradually added into the *C. comosa* extract mixture under continuous stirring. The resulting mixture was then adjusted its pH to 4.5 ± 0.1 with sodium hydroxide solution. The stirring was performed till obtaining a homogeneous gel.

In the case of the gels prepared with the mixture of HPMC and PCP, the HPMC was dispersed in hot water (90 °C), whereas PCP was dispersed in room temperature water. The resulting HPMC and PCP dispersions were then thoroughly mixed, followed by addition into the *C. comosa* extract mixture and adjusting the mixture pH to 4.5 ± 0.1 by sodium hydroxide solution. Continuous stirring was carried out to yield a homogeneous gel.

All the *C. comosa* gels were sealed in a tight, light-resistant container and kept at room temperature for approximately 24 h before evaluations.

### 2.4. Incompatibility between C. comosa Extract and Gel Components

The incompatibility between *C. comosa* extract and gel components was investigated using Attenuated total reflection (ATR)-Fourier transform infrared (FTIR). The ATR-FTIR characteristics, in the transmission mode, through the 600–4000 cm^−1^ of *C. comosa* extract, mucoadhesive polymers, glycerol, *C. comosa* gels, and the gels without *C. comosa* extract (placebo gels) were assessed using a BRUKER TENSOR 27 FTIR spectrometer (Bruker Corporation, Billerica, MA, USA) coupled with a zinc selenide ATR crystal. A background spectrum was performed using the dry and clean ATR crystal in the air. Each spectrum was determined at a resolution of 4 cm^−1^ with an average of 32 scans. After each sample determination, the ATR crystal surface was cleaned by gently wiping it down with a paper towel wet with absolute ethanol. All the ATR-FTIR spectra were captured using Bruker OPUS software (Bruker Corporation, Billerica, MA, USA).

### 2.5. Physicochemical Characteristics of C. comosa Gels

#### 2.5.1. Appearance and pH

The physical appearance and homogeneity of *C. comosa* gels were visually inspected. The gel pH was recorded using the flat surface pH electrode equipped with SevenCompact™ S220 (Mettler-Toledo GmbH, Greifensee, Switzerland).

#### 2.5.2. DA Contents

A known weight of *C. comosa* gels (0.10 g) was mixed with 20 mL acetonitrile in a 50 mL centrifugation tube. The mixture was vortexed for 5 min and ultrasonicated (Model LUC-405, Daihan Labtech Co. Ltd., Gyeonggi-do, Republic of Korea) for 60 min to extract DA1 and DA2 from the gel. The mixture was then filtered through a 0.45 μm syringe filter (CNW^®^ technologies, Shanghai, China), and the obtained filtrate was analyzed for DA content using HPLC assay.

#### 2.5.3. Rheological Properties

Rheological analysis of *C. comosa* gels was evaluated using a Haake Mars rheometer (Thermo Fisher Scientific, Wilmington, DE, USA) with a 60 mm parallel steel cone and plate geometry, separating with a 0.3 mm gap. The gel samples were carefully applied to the lower plate, providing that sample shearing was diminished, and allowed to equilibrate for 5 min at 37 ± 0.5 °C prior to analysis. In continuous shear (flow) analysis, the upward flow curves for each formulation were measured over shear rates ramped from 1 and 500 s^−1^. All measurements were carried out for at least three replicates.

To quantitatively analyze flow behavior, the Power law represented as Equation (1) was applied [30].

(1) $$\tau =k\cdot \gamma^{n}\;$$

(2) logτ=logk+nlogγ

where *τ* refers to shear stress and *γ* refers to shear rate. To clarify the flow index (*n*) and the consistency index (*k*) for different gels, the slope and intercept of the plot between the log value of *τ* and log *γ*, Equation (2), were determined and calculated.

The yield stress was determined indirectly by extrapolating a flow curve (shear stress versus shear rate) from very low shear rates to zero shear rates [30,31].

Based on the viscosity–shear rate curve, the viscosity values corresponding to a rate of shear of 5 s^−1^ were determined in order to evaluate the effect of gelling agents on the viscosity of *C. comosa* gels.

#### 2.5.4. Mechanical Properties

The mechanical properties of *C. comosa* gel were determined using the TA.XTplus Texture Analyzer equipped with a 5 kg load cell [34,35]. The *C. comosa* gel was gently packed, to avoid air bubble formation, into the sample holder to a height of 40 mm, and positioned centrally underneath a probe. The compression mode test was performed by using the 10 mm diameter analytical probe that compressed the tested gel, up and around the edge of the disc, twice. The probe was lowered at a defined speed (2 mm/s) into and through the gel for 15 mm depth. Contact was detected by a triggering force of 1 mN. After the first compression, the probe was moving up to the gel surface (2 mm/s) and the second compression was started after 15 s of awaiting time. The resulting force versus time curve (Figure S1a), was recorded and used to calculate the mechanical parameters including hardness, compressibility, cohesiveness, and elasticity.

#### 2.5.5. Extrudability

The work needed to force *C. comosa* gel out through a small opening was assessed using a TA-XTplus texture analyzer (Stable Micro Systems, Godalming, Surrey GU7 1YL, United Kingdom) coupled with forward extrusion cell with the outlet diameters of its sample container base discs of 3 mm diameter. The test was performed under compression mode. The tested *C. comosa* gel was gently packed, to avoid air bubble formation, into the cell to a height of 40 mm. The piston disc probe (50 mm diameter) was lowered at a 1.0 mm/s speed down for a 35 mm distance to extrude a gel. The compression force required for a piston disc to extrude a product through a standard-size outlet in the base of the sample container was recorded and plotted with the distance. The area under the force–distance curve (Figure S1b) was calculated and referred to the extrudability.

#### 2.5.6. Mucoadhesive Properties

Mucosa preparation: The fresh female genital organ from slaughtered pigs was given as waste from a local slaughterhouse (Khon Kaen, Thailand). The vaginal area was isolated, thoroughly washed, and the underlying connective and adipose tissues were gently cut off. The resulting vaginal mucosa, approximately 2 mm thick, was cut into 25 × 25 mm pieces, kept in the pH 5.5 simulated vaginal fluid (SVF) at 4 ± 2 °C, and employed within 24 h [18].

To determine the mucoadhesive properties of *C. comosa* gels, TA.XT.Plus Texture Analyzer (Stable Micro Systems, Godalming, Surrey GU7 1YL, UK) coupled with a 50 N load cell was utilized (Figure S2) [18,36]. The *C. comosa* gels and the porcine vaginal mucosa were incubated at 25 ± 1 °C. To start the mucoadhesion test, the porcine vaginal mucosa was integrated with an upper flat-faced 10 mm diameter cylinder probe (P10 Perspex) in the direction that the mucosa side facing out, being ready to adhere the *C. comosa* gel. The tested gel (10 g) was filled in the 50 mm diameter cylindrical shape container. The surface of the packed gel was carefully smoothened to yield a flat and bubble-free surface. The mucosa-attached probe was moving down with a predetermined speed of 1 mm/s onto the gel surface. The adhesion was set with an initial contact force of 0.1 N and an adhesion time of 120 s. Then, the mucosa-attached probe was removed at a 0.5 mm/s rate. The resulting detachment force versus the time curve was recorded (Figure S1c). The Texture Exponent 32 software (Texture Technologies Corp, Hamilton, MA, USA) was used to determine the maximum detachment force (*F_max_*) and the work of adhesion (*W_ad_*).

#### 2.5.7. In Vitro Release

In vitro release of DAs from the *C. comosa* gels was conducted under sink conditions using the modified Franz diffusion cell with a diffusion area of 2.01 cm^2^ and receiver volume of 14.0 ± 0.2 mL. The diffusion cells were attached to a water bath (WiseCircu^®^ WCB, Daihan Labtech Co. Ltd., Gyeonggi-do, Republic of Korea) maintained at the temperature of 37.0 ± 0.5 °C. A receiver medium, 50% *v*/*v* PEG 400 in pH 5.5 SVF, was loaded into a receiver compartment where its hydrodynamic was preserved by a magnetic stirring (digital magnetic stirrer, VELP Scientifica Srl, Usmate, Italy). The hydrated polyamide membrane (0.45 µm pore size, 25 mm diameter, Sartorius™, Fisher Scientific, Waltham, MA, USA), as the supported membrane, was mounted between the donor and receiver compartment with a clamp. Then, 0.6 g of *C. comosa* gel was carefully placed on the membrane on the donor side, which was tightly covered with paraffin film. At predetermined times (0, 2, 4, 6, 8, 12, 24, 36, 48, 60, and 72 h), a 0.8 mL of receiver medium was collected from the receiver compartment and the fresh 50% *v*/*v* PEG 400 (0.8 mL, pre-warmed at 37 °C) was replenished after each sampling. The content of DA released was determined by using an HPLC assay.

*Release data analysis:* The cumulative amount of DA released per area was computed and plotted against time. The cumulative release results were fitted to zero-order, Higuchi, and Korsmeyer–Peppas models, which are defined by the following equations.

(3) $$M_{t}=K_{0}\cdot t$$

(4) $$M_{t}=K_{H}\cdot t^{1/2}$$

(5) MtM∞=KKP·tn 

where *M_t_* refers to the amount of DA released at time *t*. *M_t_*/*M*_∞_ refers to the fractional DA released at time *t*. *K*_0_, *K_H_*, and *K_KP_* are the zero-order, Higuchi, and Korsmeyer–Peppas rate constants, respectively. To interpret the release mechanism, the *n,* denoted as the release exponent, was determined from the slope of the plot between the log value of MtM∞, where the fractional DA released ≤60%, and log *t*, Equation (5) [37,38,39].

### 2.6. Stability of C. comosa Gels

The physical and chemical stabilities of the selected *C. comosa* gel formulations were investigated according to the ICH guidelines [40]. The freshly prepared *C. comosa* gel was packed in a 20 g collapsible aluminum tube and kept under long-term (5 ± 3 °C) for 12 mo and accelerated (30 ± 2 °C with 75 ± 5% relative humidity (RH)) conditions for 6 mo. After 6 and 12 mo of storage, the aged *C. comosa* gel was withdrawn and investigated for percentages of DA remaining and the physicochemical characteristics, namely, appearance, pH, and viscosity. The percentage viscosity, which refers to the values of viscosity relative to that of its fresh gel, was calculated.

### 2.7. HPLC Determination of DAs

The DA amount was quantified using a validated HPLC protocol previously reported by Tunpanich et al. [18]. An Agilent 1260 Infinity LC system (Agilent Technologies, Inc., Santa Clara, CA, USA) equipped with an autosampler and a diode array detector running at 260 nm was utilized. The chromatographic separation was conducted on a ZORBAX Eclipse Plus column (octadecyl, 3.5 μm particle size, 100 mm length, 4.6 mm inner diameter; Thermo Fisher Scientific, Wilmington, DE, USA) set at 30 °C with a mixture of water and methanol (gradient ranged from 30:70 to 40:60, 0.9 mL/min flow rate) as a mobile phase. The DA1 and DA2 were properly eluted at the 23.7 and 25.5 min retention times, respectively. The separation procedure demonstrated excellent linearity (R^2^ > 0.9999) throughout the determination range of 1–50 μg/mL.

### 2.8. Statistical Analysis

The experiment results are presented as the mean ± S.D. One-way analysis of variance (ANOVA) was used to test the statistical significance of differences among groups. The significant difference in the means was further determined by using Tukey’s post hoc test. The statistical tests were operated using the SPSS program for Windows software (Version 17.0, Released 2008, Chicago, IL, USA: SPSS Inc.). The statistical significance was set at *p* < 0.05.

## 3. Results and Discussion

### 3.1. C. comosa Gel Characteristics and pH

In this study, the mucoadhesive gels of *C. comosa* ethanolic extract were fabricated using the mucoadhesive polymers—PAA (C980 and PCP), cellulose derivatives (HPMC K15M and HPMC K100M), and a mixture of PAA:cellulose derivatives (2:1 PCP: HPMC)—as a gel-forming and mucoadhesive agent. Because of the hydrophobicity of the DAs (Figure 1) and other hydrophobic components in *C. comosa* extract [41], *C. comosa* extract had to be homogeneously blended with the high HLB surfactant (polysorbate 80) as a wetting agent before mixing with other ingredients, to obtain the uniformity mixture with the aqueous polymeric dispersion, especially for the HPMC K15M-based formulations. The *C. comosa* gels were also composed of glycerol as a humectant and methylparaben as a preservative. Sodium hydroxide solution was used to adjust the gel pH to the range of 4.5 ± 0.1. In the present study, all the *C. comosa* gels were designed to possess a moderately acidic pH to conform with the normal vaginal pH ranges (3.8–5.0) for women [42]. It is known that maintaining the acidic pH balance of the vaginal tract is vital for common vaginitis prevention.

**Figure 2:**
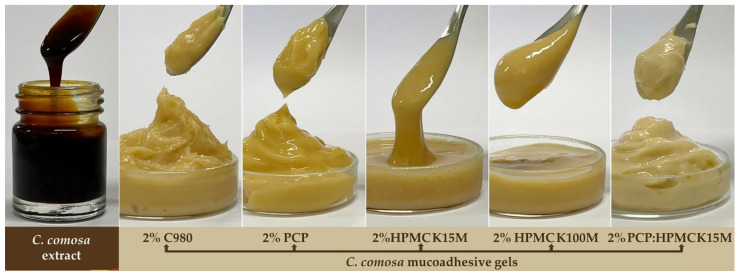
Macroscopic characteristics of *C. comosa* extract and *C. comosa* mucoadhesive gels prepared from various mucoadhesive polymers.

Figure 2 illustrates the macroscopic characteristics of *C. comosa* gels as compared with the *C. comosa* extract. All the *C. comosa* mucoadhesive gels were light brown, opaque, homogeneous viscous gels with a characteristic odor of *C. comosa* extract, whereas the placebo gels—the gels without *C. comosa* extract—were colorless, almost clear to clear gels (Figure S3). The PCP:HPMC-based *C. comosa* gels appeared to be a little more yellow-white than the others. The homogeneity and consistency of *C. comosa* extract with the gel bases was in the order of PAA (C980 and PCP) > HPMC K100M > HPMC K15M. It should be noted that *C. comosa* extract can be easily incorporated into PAA-based gels regardless of polysorbate 80 utilization.

Different mucoadhesive polymers yielded different viscosity gels, among which HPMC K15M yielded the mucilaginous characteristic. The viscous gel matrix was caused by the continuous polymer network, physically structured through the physical entanglements and/or secondary interactions between polymer chains, whereupon an aqueous phase, as well as *C. comosa* extract, was captured. In the case of PAA-based gels, the jelly-like gels were obtained through neutralization, which induced the ionization of carboxylic acid groups, uncoiling the PAA chains through electrostatic repulsion [19,30]. All the *C. comosa* gels had a pH in the designed range of 4.4–4.6. The actual loading of DA1 and DA2 in *C. comosa* gels were determined and found to be 0.47–0.49% of each DA, which were corresponding to 94.1 ± 3.3 to 97.4 ± 2.75% and 96.8 ± 2.9 to 99.6 ± 3.6% of theoretical loading of DA1 and DA2, respectively.

### 3.2. Incompatibility Study

**Figure 3:**
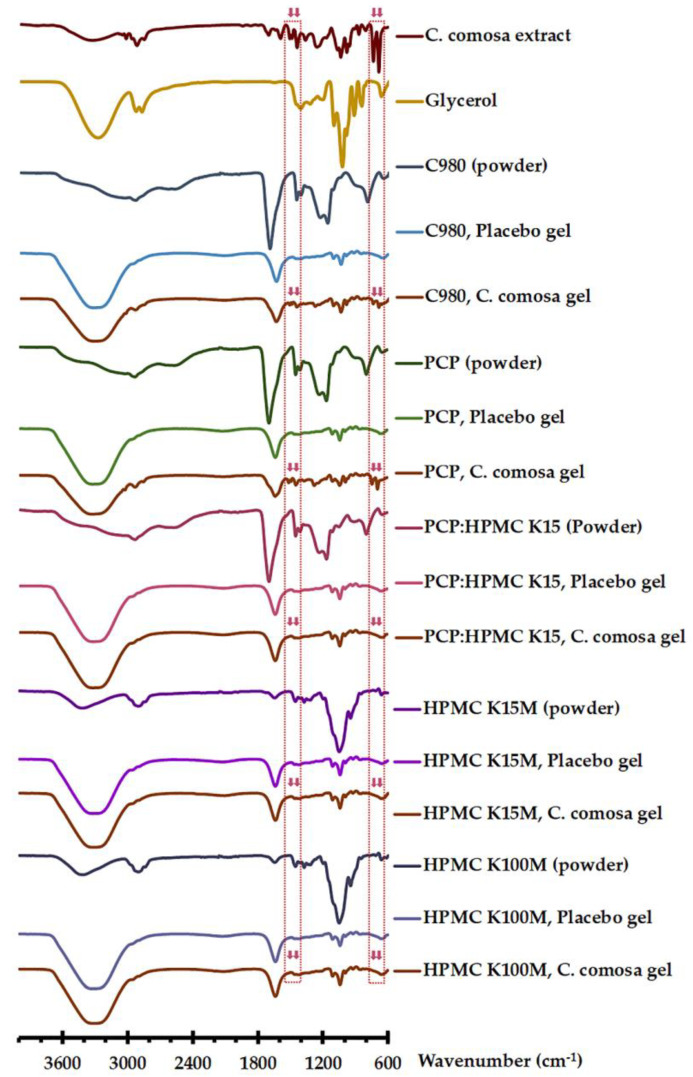
ATR-FTIR of *C. comosa* extract, glycerol, mucoadhesive polymers, placebo gels, and *C. comosa* gels.

The compatibility between *C. comosa* extract with mucoadhesive polymers as well as gel compositions was assessed using ATR-FTIR. As presented in Figure 3, the FTIR spectrum of *C. comosa* extract comprised the bands at 3333 cm^−1^ (O-H stretching), at 2800–3100 cm^−1^ (aliphatic C-H stretching), 1601 cm^−1^ (C=C stretching), and at 900–700 cm^−1^ (C-H bending) [12,14,18]. C980 and PCP displayed the broad absorption bands for O-H and C-H stretching at 3600–2800 cm^−1^, a strong band for C=O stretching at 1697 cm^−1^, bands for C=O and C-O stretching at 1450 and 1166 cm^−1^, respectively. For HPMC K15M and HPMC K100M, the major absorption bands observed were a band at 3415 cm^−1^ due to O-H and stretching, at 2900 cm^−1^ due to C-H stretching, and a strong band at 1051 cm^−1^ assigned to C-O stretching. The FTIR spectrum of PCP and HPMC K15 physical mixture (2:1 ratio) revealed distinct bands comparable to those of PCP [18]. The FTIR spectrum of glycerol is recorded to be of O-H stretching (3282 cm^−1^), aliphatic C-H stretching (2931 and 2880 cm^−1^), and H-O-H bending (1640 cm^−1^) [36]. The ATR-FTIR spectrum of placebo gels, irrespective of the mucoadhesive polymers, displayed strong absorption bands at 3315 cm^−1^ (O-H stretching) and a band at 1637 cm^−1^ (H-O-H bending). It should be noted that the intermolecular interaction between PCP and HPMC was not observed in the FTIR spectra of *C. comosa* and placebo gels. These could probably be concealed by the strong H-O-H bending band due to glycerol and water molecules. According to the reports published previously, the hydrogen bonding between the –COOH group of PAA polymers (carbomer [43] and PCP [44]) and –OH group of HPMC, while absence in the solid state [18,43], could be formed under the hydrated conditions—overnight-soaked carbomer 71G-NF:HPMC K15M tablets [43] and PCP:HPMC K15M films [44].

The FTIR spectra of *C. comosa* mucoadhesive gels prepared with C980 and PCP displayed the definite bands corresponding to those of placebo gels and *C. comosa* extract at approximately the equivalent positions. For *C. comosa* gels based on C980 and PCP, the characteristic bands of *C. comosa* extract at around 650–750 and 1400–1550 cm^−1^ were clearly observed. These indicate the absence of considerable incompatibility among *C. comosa* extract and the components in the C980-based and PCP-based gel formulations. Interestingly, in the case of HPMC-based and PCP:HPMC-based gels, the disappearance of the FTIR characteristic bands in the 650–750 and 1400–1550 cm^−1^ regions of *C. comosa* extract was observed. These suggested the possible interaction between HPMC and the components of *C. comosa* extract. HPMC is a hydrophilic polymer possessing the ability to form a polymer-type complex [45]. Nevertheless, in addition to DAs, *C. comosa* extract contains also numerous phytochemical compounds, namely, diterpenes, curcucomosins, flavonoid glycosides, curcucomosides, and sesquiterpines [41,46]. To prove if the undesirable incompatibility between DAs and HPMC occurred, the influence of HPMC on the release behaviors and chemical stability of DAs should be further investigated.

### 3.3. Rheological Properties

**Figure 4:**
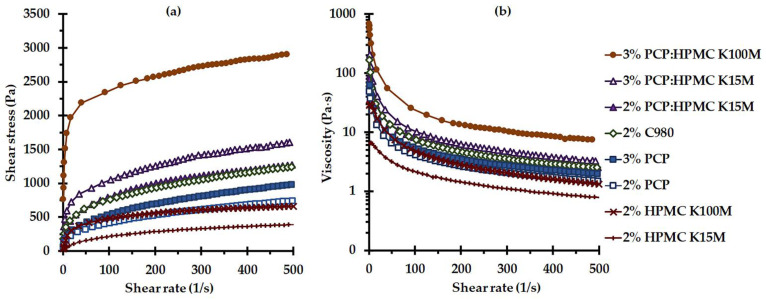
Flow rheograms (**a**) and viscosity–shear rate curve (**b**) at 37 °C of *C. comosa* mucoadhesive gels prepared from various mucoadhesive polymers.

Rheological behaviors directly influence the gel performance such as application, spreadability, and retention time in the vaginal cavity. The rheological properties of *C. comosa* gels were assessed using the basic destructive (flow) technique. This destructive method is a commonly used technique to determine the rheological performance of vaginal gels and to provide information regarding in what manner the gels behave under a shear stress circumstance owing to the motions of the vaginal surfaces, and gravitational and capillary flow [30]. The flow behaviors of *C. comosa* gels were determined at 37 °C to mimic the rheological performances of these gels in the vaginal cavity. Their rheograms, which illustrated the relation between the shear rate and shear stress, are presented in Figure 4a. All the *C. comosa* gels exhibited non-Newtonian (pseudoplastic) flow revealing a shear-thinning appearance, and a high viscosity at a low shear rate, which subsequently decreases with shear.

Flow behavior in the shear-thinning region was well characterized by the power law, which is a practical equation most utilized for pseudoplastic material expression. As presented in Table 2, each curve demonstrates a power law relationship between shear stress (*τ*) and shear rate (*γ*) with a coefficient of determination of 0.95–0.99. The flow behavior index (*n*)*,* as well as consistency index (*k*)*,* differed in each gel formulation depending upon the type and concentration of mucoadhesive polymer. The degree of shear-thinning behavior was determined by the *n* value [30]. The *C. comosa* gels have *n* values of less than one, within the range of 0.177–0.376, confirming the shear-thinning phenomena. The closer the *n* value is to zero, the larger the shear-thinning behavior of *C. comosa* gels. Blending PCP with HPMC yielded a more pronounced pseudoplastic behavior gel when compared with the neat PCP-based or HPMC-based gels. This might be associated with the intermolecular complexation among PCP and HPMC [18,43,44]. It has been reported that the stronger gels exhibit a lower *n* value due to the increase in the attraction bond between adjacent molecules, which enhances the existence of temporary entanglement associations [47]. With regards to the *k* value, it was noticed that the higher the *k* value, the more viscous consistency of the *C. comosa* gels with a lower *n* value.

Yield stress can be defined as the minimum stress that has to be placed to start the flow. As listed in Table 2, the yield values of *C. comosa* gels differ between formulations ranging from approximately 1 to 237 Pa. The extent of yield stress indicates the intensity as well as the strength of the closed pack between adjacent molecules of the gel structure [47]. Yield stress denotes the residence time and the spreadability of the gels in the vaginal cavity under low-force experience [31]. The higher yield stress of PCP:HPMC-based gels suggested their long residence time in the application site, while a too-high yield value of 3% PCP:HPMC K100 may limit its spreadability throughout the vaginal mucosa.

The nonlinear response to shear stress of *C. comosa* gels was caused by the change in polymeric structure upon shear stress exposure. The investigated gels might contain a highly entangled polymer in a relaxed state, which, as a whole and/or polymer chain segments, would disentangle and then align themselves in the direction of shear. The solvent located in the coil structure was released. The number of entanglements between polymer chain segments and side chains was decreasing with shear stress. Under high shear stress, polymer chains were becoming highly mobile and started to slide past one another due to the inter- and intra-molecular bond destruction, offering low resistance to flow, and the apparent viscosity was decreased [30,47].

Pseudoplastic behavior is considered a preferable characteristic for vaginal gels. A gel exhibits considerable viscosity at low shear representing the initial physical stability and a high potential to retain at the applied location, while a decrease in an apparent viscosity under high shear stress provides the ease of administration as well as spreadability on the application.

The shear viscosity at 37 °C of the *C. comosa* gels as a function of shear rate is presented in Figure 4b. Their curve starts with a yield value and their viscosity decreases with an increase in shear rate. The viscosity of the *C. comosa* gels at low shear rates (5 s^−1^) was also determined, their viscosity varies from 6.2 to 335.4 Pa·s. It should be noted that the presence of 3% *w*/*w C. comosa* extract did not affect the flow behavior of the gels; however, it slightly increased the viscosity values for all formulations when compared with placebo gels. The viscosity at a low shear rate could be used to indicate the viscosity during resting in the vaginal cavity. Similar to the yield stress values, the viscosity of the gels increased with polymer concentration. C980 yielded a higher viscous gel as compared with PCP. The viscosity of 3% PCP:HPMC K100M was found to be the highest, while the HPMC K15M-based gels showed the lowest viscosity among all the formulations. The low viscosity may result in the prompt loss of *C. comosa* gel from the application site, while the high viscous gels might exhibit poor spreadability on the vaginal mucosa after application.

### 3.4. Texture Profile Analysis and Extrudability

Vaginal gels shall possess suitable mechanical characteristics, namely, low hardness, ease of application, mucoadhesive, and retention prolongation at the application site. Therefore, *C. comosa* gels were investigated as per their mechanical properties using a texture profile analyzer. Texture profile analysis contributes the details regarding the response to the external compressional force and the ability to generate the gel structure deformations, either reversible or irreversible [34,35]. Table 3 presents the compressibility, hardness, and cohesiveness of the *C. comosa* gels. The hardness of *C. comosa* gels ranged from 0.021 to 0.310 N, depending on the type and concentration of mucoadhesive polymers. For the gels prepared from the same concentration of neat mucoadhesive polymer, the hardness of the gels was in the order of HPMC K15M < HPMC K100M < PCP < C980 (*p* < 0.05). Blending HPMC K15M with PCP significantly increased the gel hardness (*p* < 0.05). Increasing the concentration of mucoadhesive polymer enhanced dramatically the gel hardness values (*p* < 0.05). This was linked to the denser polymeric network in the gels when the higher polymer concentration was used. The compressibility of *C. comosa* gels ranged from 0.098 ± 0.002 to 1.448 ± 0.051 N·s. Analogous to the hardness values, the compressibility was determined by the type, molecular mass, and concentration of mucoadhesive polymers. These textural properties were associated well with the rheological properties of the gels. This agrees with the previous reports investigating the compression behaviors of various polymers—carbomers, hydroxyethylcellulose, polyvinylpyrrolidone, and polycarbophil [35,48]. Fresno et al. [48] found that an increase in the concentration of carbomer caused a linear increment in the hardness and compressibility of the hydroalcoholic gels. Santos et al. [35] have demonstrated that the hardness and compressibility of the emulsion-based systems containing propolis increased with the carbomer 934P content.

The *C. comosa* gels exhibited good cohesiveness ranging from 0.914 ± 0.037 to 1.236 ± 0.052 of which the HPMC-based gels had the highest cohesiveness (*p* < 0.05). The PAA-based gels exhibited comparable cohesiveness, which was not influenced by the polymer concentration or the presence of HPMC in the systems (*p* > 0.05).

Hardness, the so-called firmness, refers to the maximum compression force required to reach a given deformation, whereas compressibility refers to the work needed to deform the gels during the first compression. Hardness and compressibility manifest the work or stress needed to withdraw the *C. comosa* gels from a container as well as the applicability to the target site, producing a homogeneous layer and providing the product comfort. Low hardness and compressibility values signify the ease of gel removal from the container and its spreadability over the application site. Cohesiveness refers to the proportion of the area under the force–time curve generated during the second compression over that formed during the first compression cycles [20]. Cohesiveness contributes to the details regarding the effects of repeated shearing stresses on the structural properties of formulations and is associated with structural recovery after facing the sequential shear stresses. Therefore, the high cohesiveness values suggest that the gel system has a high structure arrangement and behavior at the application site [34,35].

It should be noted that all *C. comosa* gels exhibited comparable elasticity with values ranging from 0.999 ± 0.007 to 1.019 ± 0.009 mm (data not shown). These indicate that the rate at which the deformed gels recovered to their original structure of *C. comosa* gels was not affected by the type, molecular mass, and concentration of mucoadhesive polymers. Additionally, it was found that the textural properties of *C. comosa gels* were comparable to those of placebo gels, indicating that the incorporation of 3% *C. comosa* extract did not affect the mechanical behaviors of the investigated mucoadhesive gels.

Extrudability was investigated and the results are listed in Table 3. As seen, the work needed to expel the gels through a small-opened hole (3 mm diameter) depends on the type of mucoadhesive polymers and was in the order of HPMC K15M ~ PC*P* < HPMC K100M < C980 (*p* < 0.05). Blending PCP with HPMC dramatically enhanced the extrudability values as compared to the gels prepared with neat polymers (*p* < 0.05). The extremely high extrudability of 3% PCP:HPMC K100M might be unfavorable for the practical administration of gels with a syringe. Interestingly, increasing the polymer concentration from 2% to 3% had no significant effect on the extrudability of PCP-based gels, whereas raised the extrudability value of PCP:HPMC K15M for about 1.5 folds. This extrudability parameter indicates the ease of administering the *C. comosa* gels with an applicator or syringe. It has been claimed that the work to discharge the semisolid formulations through an applicator (so-called syringeability) to periodontal pockets sites should be within 20–380 N·mm for injectable application via syringe systems [49]. Nevertheless, for semisolid preparations intended for vaginal application, the limitation of syringeability value has not been precisely determined.

### 3.5. Mucoadhesiveness

**Figure 5:**
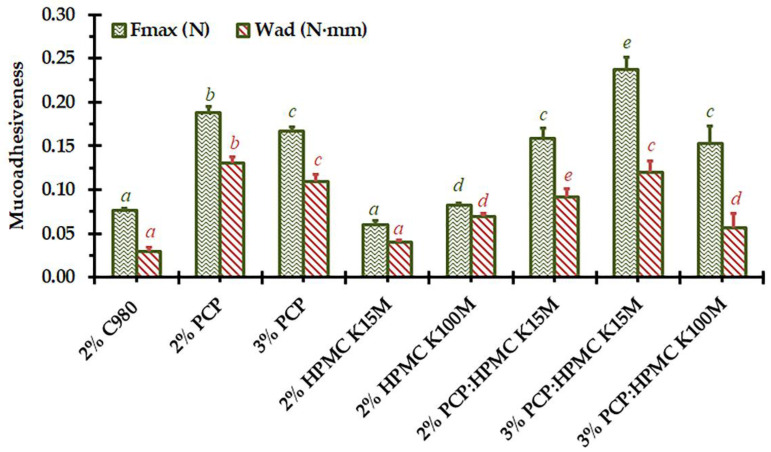
Mucoadhesiveness, in terms of maximum detachment force (*F_max_*, N) and work of adhesion (*W_ad_*, N·mm), of *C. comosa* mucoadhesive gels. Means of six determinations (±SD) values with different superscript letters are significantly different (*p* < 0.05, one-way ANOVA followed by Tukey’s post hoc multiple comparisons).

The mucoadhesive ability of a vaginal gel formulation is considered an essential variable to achieve prolonged retention and release duration at the application site. The adhesive strength, in terms of maximum detachment force (*F_max_*) and work of adhesion (*W_ad_*), of the *C. comosa* gels to the porcine vaginal mucosa was therefore conducted. As presented in Figure 5, the difference in mucoadhesiveness of *C. comosa* gels was found, with their *F_max_* and *W_ad_* ranging from 0.060 to 0.237 N and 0.029 to 0.131 N·mm, respectively. The ranking of mucoadhesiveness of *C. comosa* gels prepared with 2% mucoadhesive polymer was HPMC K15M ~ C980 < HPMC K100M < PCP (*p* < 0.05). Blending of PCP with HPMC K15M yielded a significant increase in mucoadhesiveness than those of HPMC K15M gels. An increase in the PCP concentration from 2 to 3% decreased the mucoadhesiveness, while the mucoadhesiveness of PCP:HPMC K15M increased with the polymer concentration increment.

It is known that mucoadhesion, the adhesion between mucoadhesive formulations and mucin glycoprotein in the mucus layer, arises after intimate contact between these two surfaces. The swelling of the gels upon contact with the mucosal membrane induces the relaxation of the entangled or twisted polymeric chains, enhancing the chain mobility for interdiffusion/interpenetration with a biological substrate. The physical entanglement followed by the secondary non-covalent interactions—hydrophobic, hydrogen, and van der Waals—between substrates occurs and strengthens the mucoadhesion [26,50]. Concerning the mechanism of mucoadhesion, numerous physicochemical properties of mucoadhesive polymers—molecular weight and chain length, functional group contribution, and degree of cross-linking—and formulation properties—pH, polymer concentration—as well as rheological and mechanical properties, exhibit significant influence on the extent of polymer/mucin glycoprotein interaction. The stronger mucoadhesion of HPMC K100M over that of HPMC K15M was related to its higher molecular mass, which is requisite for entanglement with mucin glycoprotein [26]. The inferior mucoadhesive ability of HPMC-based compared to PAA-based gels was consistent with the previously reported results [18]. HPMC is a non-ionic polymer with the –CH_3_ or –CH_2_CH(CH_3_)OH functional groups, whereas C980 and PCP are the high molecular weight PAA possesses numerous –COOH in their structure [26,28,50]. The considerable proton-donating carboxylic group content of PAA causes better hydrogen bonding ability and, thus, mucoadhesiveness compared to HPMC. The stronger mucoadhesiveness of PCP:HPMC-based than those of HPMC-based gels was associated with the mucoadhesive capability of PCP and probably with the synergistic impact on the mucoadhesion due to the intermolecular complexation among PCP and HPMC [18,43,44]. The excellent mucoadhesiveness of PCP-based gels than the others may be associated with their ability to form strong hydrogen bonds and sufficient chain flexibility. The better chain flexibility/mobility, the better the interdiffusion and interpenetration through the mucin network. PCP is a lightly cross-linked PAA, while C980 is a highly cross-linked PAA [28]. A high degree of cross-link density decreases the polymeric chain mobility, which further reduces the effective chain length, mucin interpenetration, and eventually the mucoadhesive strength [26]. Additionally, the moderate rheological and mechanical properties of PCP-based gels as compared to those of C980-based gels may confer better interdiffusion and interpenetration of the mucoadhesive polymer through the mucus layer. The different influence of polymer concentration increment on the mucoadhesiveness of PCP-based and PCP:HPMC K15M-based gels was probably related to their difference in the optimum concentration. It has been demonstrated that, for semisolid preparations, each mucoadhesive polymeric system possesses an optimum concentration. Excessive concentration beyond the optimum contains the coiled and insufficient hydrated molecules, limiting the number of available polymer chains for mucin interdiffusion and interpenetration, which eventually diminishes the mucoadhesiveness [26,27].

### 3.6. In Vitro DA Release

**Figure 6:**
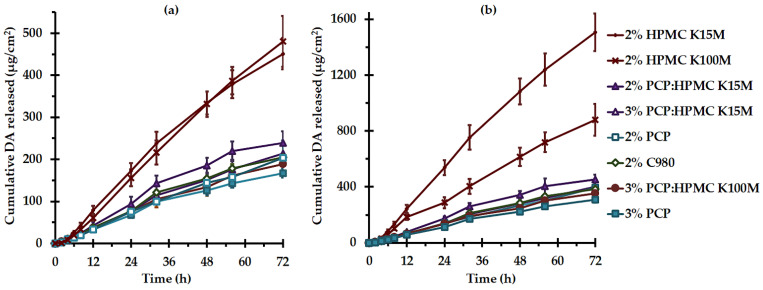
In vitro release behaviors of DA1 (**a**) and DA2 (**b**) from *C. comosa* mucoadhesive gels (mean ± SD, *n* = 4).

The release behavior of active compounds from a gel matrix is a prerequisite for mucosal permeation. The DA release patterns from the *C. comosa* gels were therefore examined in vitro via a static diffusion cell. Because DA compounds are practically insoluble in the aqueous medium (Figure 1), 50% *v*/*v* PEG 400 was incorporated into SVF pH 5.5 to obtain the sink condition. The solubility of DA1 and DA2 in SVF pH 5.5 (at 37 °C) was found to be 4.3 ± 1.8 and 9.2 ± 2.9 μg/mL, respectively. The presence of 50% PEG 400 in the SVF yielded the DA solubility enhancement of approximately 63- and 84-fold for DA1 and DA2, respectively, compared to those in SVF. PEG 400 has been proven as an efficient cosolvent to enhance the solubility of DAs in the receiver medium [15,16,51]. The DA release was examined at a predetermined time of up to 72 h and their cumulative release profiles are presented in Figure 6. All *C. comosa* gels exhibited the DAs release over a period of 72 h in a sustained release pattern. It can be seen that the HPMC-base gels provided the highest DA release compared to the other *C. comosa* gels. The rate and extent of DA release from the *C. comosa* gels were found to be retarded by the presence of PAA. It is obvious that the release of DA2 was significantly higher than that of DA1 (*p* < 0.05), irrespective of the type and concentrations of the mucoadhesive polymer in the formulations. This phenomenon was associated with the higher solubility of DA2 in the medium (*p* < 0.05). This is in agreement with the previous report regarding the DA release from *C. comosa* mucoadhesive vaginal tablets [18].

To assess the pattern of DA release from the *C. comosa* gels, the release data were fitted through the typically utilized kinetic equation models, zero-order and Higuchi. As presented in Table 4, the release of DA1 and DA2 from all *C. comosa* gels was best explained by the zero-order of which their coefficient of determination values was ≥0.99, whereas the coefficient of determination values obtained from the Higuchi model was ≥0.97. These suggest that the rate of DA release from the *C. comosa* gels was constant and did not depend upon the DA concentration. The rates of DA1 release, K_0_, from 2% HPMC K15M and 2% HPMC K100M were comparable, whereas 2% HPMC K100M yielded a significantly lower rate of DA2 release than that from 2% HPMC K15M. This might be associated with the lower solubility of DA1 than DA2, which caused the less sensitivity of DA1 release to the higher molecular weight HPMC three-dimensional structure. It is interesting to note that the release of DA2 from 2% HPMC K15M was almost complete with the Q_72h_ of 3 mg (Table 4) or approximately 100% of the theoretical loading. For neat PAA gels, the K_0_ of both DAs from C980-based and PCP-based *C. comosa* gels was comparable regardless of the polymer concentration. A combination of PCP with HPMC could significantly retard the DA release when compared with neat HPMC-based gels (*p* < 0.05). The DA release rate was decreased with the PCP:HPMC concentration (*p* < 0.05).

It is generally acknowledged that the principal mechanisms determining the release from the hydrogel-based drug delivery systems are diffusion, swelling, polymer relaxation and disentanglement, and/or erosion of the polymeric matrix. Diffusion of active ingredients from the polymeric matrix may happen on a molecular level—the passage between polymer chains—or on a macroscale level—the diffusion pores in a polymer matrix. A three-dimensional macromolecular mesh networks made of reticular polymers behave as a diffusion layer proving a sustained release capability. The release behaviors could be caused by explicit or sophisticated mechanisms depending on the physicochemical properties of both the active ingredient and the polymeric matrix, as well as active ingredient-matrix interaction [37,52,53]. Based on the relative magnitude of the rates of polymer relaxation at the penetrating solvent front and the diffusion of the associated active ingredient, the release behavior can follow either a Fickian or non-Fickian pattern [54]. To further investigate the release mechanisms of DA from the *C. comosa* gels, the release data were fitted as per the Korsmeyer–Peppas model, the so-called power law, which is a semi-empirical equation useful for characterizing the controlled release of polymeric systems including hydrogels, especially when the release mechanisms are engaged with more than one phenomenon [39]. The power law plots of the *C. comosa* gel release data also showed high linearity with the coefficient of determination values of ≥0.98. The exponent of release, *n*, values derived from the slope of the plot calculated based on the Korsmeyer–Peppas equation indicate that the release mechanisms of DA1 and DA2 from *C. comosa* gels follow the non-Fickian transport, an approximate Case II (*n* ~ 1), which provide release characteristics approaching zero-order. These suggest that DA release from *C. comosa* gels is controlled by swelling and involves polymer relaxation and chain disentanglement [39,54].

A non-Fickian mechanism transport generally occurs with the glassy (vitreous) polymeric system having the glass transition temperature beyond the experimental temperature. In this situation, the mobility of polymer chains is insufficient to allow the penetration of water molecules into the system core [39,54,55]. Case II transport is a particular situation of non-Fickian transport where the solvent front is expanded at a relaxation-controlled steady rate. Solvent diffusion through the gelled region occurs at a faster rate as compared with the relaxation process of the gel-vitreous polymeric interface, limiting the inner penetrating of water molecules. The gelled region possesses greater solvent diffusivity and contains a water concentration level in the equilibrium equal to the external medium, while a vitreous region has almost no penetrating solvent. Lastly, a rapid increase in water absorption rate caused by the expansion forces of swollen gel in the vitreous nucleus might occur. In such a case, Super Case II transport (*n* > 1), an intense form of non-Fickian transport, is developed. For Super Case II transport, the solvent diffusion occurs at a faster rate, accelerating the solvent penetration. The tension generated during the movement of the gel–vitreous interface causes polymer breakage [39,55].

The differences in the release rate and extent between HPMC-based and PAA-based *C. comosa* gels may be associated with the difference in the rate of polymer relaxation and chain disentanglement caused by the molecular structure of the polymer. HPMC K15M and HPMC K100M are soluble methyl cellulose ethers with molecular weights of 750 and 1150 kDa, respectively [56]. C980 and PCP are the high molecular weight PAA cross-linked with allyl pentaerythritol (C980) or divinyl glycol (PCP) with the molecular mass of 7 × 10^2^–4 × 10^6^ and 3.5 × 10^6^ kDa, respectively [28]. The enormous molecular size together with the interconnected crosslinks of C980 and PCP might confer a slow relaxation of the polymer chain leading to DA release retardation. This is in agreement with the previous report [18].

It is noteworthy that the rapid DA release from HPMC-based gels, as well as the comparable release of DAs from PCP:HPMC-based as compared with PCP-based gels, indicate the lack of the DA and HPMC interaction (ATR-FTIR result, Figure 3) that results in the DA release retardation.

### 3.7. Physical and Chemical Stability

Considering their rheological, mechanical, mucoadhesiveness, as well as in vitro release behaviors, characterized by moderate viscosity and mechanical properties, maximum mucoadhesive force, while providing the ability to sustain DA release, the PCP-based and PCP:HPMCK15M-based *C. comosa* gels were selected for investigating the aging effect on their physical and chemical stability. Four formulations of *C. comosa* gels (Table 5) were prepared, tightly sealed in an aluminum collapsible tube, then kept under accelerated and long-term conditions for 6 and 12 mo, respectively. *C. comosa* gels are considered to be drug products intended for storage in a refrigerator due to the degradation susceptibility of DAs to high-temperature storage [14,18,57]. Accordingly, the accelerated and long-term settings for the stability investigation of *C. comosa* gels were 5 ± 3 °C and 30 ± 2 °C/75 ± 5% RH, respectively (Climatic Zone IVb [40]).

It should be noted that the visual appearance and consistency of the investigated *C. comosa* gels were maintained throughout the stability experiment; however, a slight color fading was observed. Physical properties, namely, pH and percentage viscosity of aged *C. comosa* gels versus the fresh gels are presented in Table 5. It can be seen that the pH of the aged gels tended to be increased. Though the pH of aged gels stored under long-term condition for 6 mo was comparable to that of fresh gels (*p* > 0.05), the 12 mo long-term-aged gels and 6 mo accelerated-aged gels had significantly higher pH than the fresh gels (*p* < 0.05). The viscosity of aged gels was likely to be decreased upon storage (*p* > 0.05), especially the PCP-based gels stored under long-term condition for 12 mo during which their viscosity decreased to approximately 10% of the initial value. It becomes apparent that the gel viscosity diminishes with aging, which is caused by the elastic contraction of polymer chains [58].

The percentages remaining of DAs in *C. comosa* gels after storage are presented in Table 5. It can be seen that the percentages remaining of DA1 in the gels storage under long-term condition remained constant (*p* > 0.05), ranging from 99.1 to 100.6% and 95.5 to 100.1% for 6 and 12 mo storage. For *C. comosa* gels storage under accelerated condition, the percentages remaining of DA1 were in the range of 42.1 to 66.2% of which 2% PCP ~3% PCP < 2% PCP:HPMC K15M < 3% PCP:HPMC K15M (*p* < 0.05). Interestingly, the percentages remaining of DA2 in all aged *C. comosa* gels insignificantly increased irrespective of the storage condition and time (*p* > 0.05). The higher remaining content of DA1 and comparable DA2 content of PCP:HPMC K15M-based than those of PCP-based *C. comosa* gels suggest the absence of incompatibility between DAs and HPMC (ATR-FTIR result, Figure 3), which causes a negative impact on the chemical stability of DAs.

The significant decrease in DA1 content upon storage under accelerated condition is in line with the previous report that investigated the *C. comosa* mucoadhesive tablets based on HPMC K100M [18]. It has been demonstrated that the degradation of *C. comosa* crude extract was not only a time—but also a temperature-dependent process. After 6 mo storage under 40 °C, the percentage remaining of DA1 was 13.3 ± 0.1%. The proposed degradation processes of DA1 and DA2 were oxidation and hydrogenation or dehydration. The increase in DA2 content upon storage under high temperatures, e.g., 40 °C, was associated with the degradation of some other components contained in *C. comosa* extract into DA2 [57]. This study confirms the findings of earlier research that the mucoadhesive gel of *C. comosa* extract was a product intended to be stored in a refrigerator or under low temperatures.

## 4. Conclusions

This study was designed to investigate the mucoadhesive vaginal gels for sustained release delivery of phytoestrogen DAs from *C. comosa*. The *C. comosa* mucoadhesive gels could be successfully formulated with the investigated mucoadhesive polymers, namely, C980, PCP, HPMC K15M, HPMC K100M, and 2:1 PCP:HPMC blends. Because of the large and hydrophobic molecular structure of DAs, their release from *C. comosa* gels could be sustained, in the zero-order release manner after a predetermined lag time, from the polymeric network of the investigated mucoadhesive polymers. The Korsmeyer–Peppas model fitting suggested that the release mechanisms of DA from *C. comosa* gels follow the non-Fickian transport, an approximate Case II transport, involving the polymeric swelling—relaxation and disentanglement of the polymer chains. The stability investigation indicates that *C. comosa* gels possessed excellent physical and chemical stability for up to 12 mo under a refrigerator or low-temperature storage. Considering the rheological, mechanical, mucoadhesiveness, in vitro release, and stability of DAs, the PCP:HPMCK15M-based gels were considered as a favorable mucoadhesive vaginal gel system for *C. comosa* crude extract. Nevertheless, it should be also noted that the high mucoadhesiveness formulations can give rise to local pain and tissue damage. Future investigations regarding the local irritation and clinical efficiency of the *C. comosa* mucoadhesive vaginal gels for the treatment of vaginal dryness and/or the symptoms associated with the genitourinary syndrome of menopause shall be performed.

## Figures and Tables

**Table 1:** General formulation compositions of *C. comosa* gels.

Ingredients	Amount (% *w/w*)	Functions
*C. comosa* extract	3.0	Source of DAs
Mucoadhesive polymers *	2.0–3.0	Gelling and mucoadhesive agents
Polysorbate 80	0.1	Wetting agent
Methyl paraben	0.2	Preservative
Glycerol	15.0	Humectant, solvent of methyl paraben
4% *w/v* sodium hydroxide solution **	qs. to pH 4.5 ± 0.1	pH adjusting agent
Deionized water to	100.0	Vehicle

* Mucoadhesive polymers were HPMC K15M, HPMC K100M, carbomer (C980), polycarbophil (PCP), or a mixture of PCP and HPMC at a weight ratio of 2:1, respectively. ** 4% *w*/*v* sodium hydroxide solution was used to adjust the pH of *C. comosa* gels.

**Table 2:** Effects of mucoadhesive polymers on the rheological exponent (*n*), consistency (*k*), yield value, and low shear viscosity of *C. comosa* mucoadhesive gels (37 °C).

Mucoadhesive Polymers	Flow Behavior Parameters			Viscosity (Pa·s)
	*n* (Pa·s)	*k*	Yield Stress (Pa)	
2% C980	0.304 ± 0.007 ^a^	187.345 ± 3.617 ^a^	81.56 ± 11.23 ^a^	74.87 ± 2.40 ^a^
2% PCP	0.366 ± 0.004 ^b^	74.884 ± 1.495 ^b^	27.27 ± 3.94 ^b^	30.80 ± 0.73 ^b^
3% PCP	0.373 ± 0.006 ^b^	96.486 ± 1.411 ^c^	38.30 ± 5.56 ^c^	48.78 ± 2.16 ^c^
2% HPMC K15M	0.376 ± 0.010 ^b^	38.242 ± 2.567 ^d^	0.96 ± 0.14 ^b^	6.19 ± 0.36 ^d^
2% HPMC K100M	0.203 ± 0.003 ^c^	188.616 ± 3.562 ^a^	4.46 ± 0.62 ^b^	27.88 ± 0.82 ^b^
2% PCP:HPMC K15M	0.294 ± 0.004 ^a^	207.628 ± 2.574 ^e^	54.50 ± 6.43 ^c^	61.45 ± 1.28 ^c^
3% PCP:HPMC K15M	0.251 ± 0.004 ^d^	334.249 ± 4.415 ^f^	106.49 ± 14.43 ^a^	127.29 ± 4.80 ^e^
3% PCP:HPMC K100M	0.177 ± 0.003 ^e^	998.752 ± 12.974 ^g^	237.08 ± 29.87 ^d^	335.36 ± 18.96 ^f^

mean ± SD, *n* = 3. ^a–g^ Within columns, means with a different superscript letter are significantly different (*p* < 0.05, one-way ANOVA followed by Tukey’s post hoc multiple comparisons). *n* is the flow behavior index and *k* is the consistency index. Yield stress was determined from the y-intercept of flow rheograms. Viscosity refers to the viscosity values at low shear value, determined at a rate of shear of 5 s^−1^.

**Table 3:** Mechanical properties (hardness, compressibility, and cohesiveness) and extrudability of *C. comosa* gels at 25 ± 1 °C as a function of mucoadhesive polymers.

Mucoadhesive Polymers	Mechanical Properties			Extrudability (N·mm)
	Compressibility (N·s)	Hardness (N)	Cohesiveness	
2% C980	1.121 ± 0.065 ^a^	0.255 ± 0.011 ^a^	0.918 ± 0.101 ^a^	605.3 ± 22.3 ^a^
2% PCP	0.604 ± 0.028 ^b^	0.133 ± 0.004 ^b^	0.992 ± 0.049 ^a^	237.4 ± 12.0 ^b^
3% PCP	0.745 ± 0.020 ^c^	0.162 ± 0.002 ^c^	0.980 ± 0.036 ^a^	231.0 ± 10.7 ^b^
2% HPMC K15M	0.098 ± 0.002 ^d^	0.021 ± 0.000 ^d^	1.236 ± 0.052 ^b^	181.7 ± 17.9 ^b^
2% HPMC K100M	0.199 ± 0.010 ^e^	0.042 ± 0.002 ^e^	1.031 ± 0.045 ^c^	330.9 ± 37.2 ^c^
2% PCP:HPMC K15M	0.719 ± 0.023 ^c^	0.156 ± 0.005 ^c^	0.922 ± 0.018 ^a^	419.7 ± 27.7 ^d^
3% PCP:HPMC K15M	1.073 ± 0.030 ^a^	0.233 ± 0.006 ^f^	0.914 ± 0.037 ^a^	626.4 ± 36.0 ^a^
3% PCP:HPMC K100M	1.448 ± 0.051 ^f^	0.310 ± 0.018 ^g^	0.917 ± 0.037 ^a^	852.2 ± 68.1 ^e^

mean ± SD, *n* = 6. ^a–g^ Within columns, means with a different superscript letter are significantly different (*p* < 0.05, one-way ANOVA followed by Tukey’s post hoc multiple comparisons).

**Table 4:** Release parameters of DAs from various *C. comosa* mucoadhesive gels.

Mucoadhesive Polymers	Zero-Order	Higuchi’s	Korsmeyer–Peppas		Q_72h_ (μg)
	*K*_0_ (µg/cm^2^/h)	*K_H_* (µg/cm^2^/h^1/2^)	*K_KP_* (%/h^n^)	*n*	
DA1					
2% C980	3.34 ± 0.15 ^a^	31.05 ± 1.48 ^a^	0.177 ± 0.021	1.05 ± 0.03	411.0 ± 21.3 ^a^
2% PCP	3.15 ± 0.17 ^a^	29.21 ± 1.66 ^a^	0.148 ± 0.015	1.08 ± 0.03	410.0 ± 81.2 ^a^
3% PCP	2.72 ± 0.22 ^a^	25.44 ± 2.00 ^a^	0.163 ± 0.019	1.02 ± 0.02	335.7 ± 21.6 ^a^
2% HPMC K15M	7.43 ± 0.62 ^b^	69.14 ± 5.92 ^b^	0.368 ± 0.075	1.07 ± 0.05	906.7 ± 75.2 ^b^
2% HPMC K100M	7.49 ± 0.77 ^b^	69.17 ± 7.33 ^b^	0.320 ± 0.057	1.08 ± 0.03	965.5 ± 122.0 ^b^
2% PCP:HPMC K15M	4.08 ± 0.35 ^c^	38.04 ± 3.39 ^c^	0.213 ± 0.092	1.06 ± 0.07	480.4 ± 56.5 ^a^
3% PCP:HPMC K15M	3.28 ± 0.38 ^a^	30.50 ± 3.57 ^a^	0.170 ± 0.017	1.05 ± 0.05	429.8 ± 38.0 ^a^
3% PCP:HPMC K100M	2.82 ± 0.47 ^a^	26.34 ± 4.47 ^a^	0.200 ± 0.019	1.00 ± 0.02	380.0 ± 46.4 ^a^
DA2					
2% C980	6.11 ± 0.18 ^a^	56.69 ± 1.85 ^a^	0.295 ± 0.035	1.07 ± 0.03	775.0 ± 51.8 ^a^
2% PCP	5.82 ± 0.35 ^a^	54.09 ± 3.28 ^a^	0.259 ± 0.024	1.09 ± 0.03	791.1 ± 132.5 ^a^
3% PCP	4.76 ± 0.27 ^a^	44.38 ± 2.44 ^a^	0.259 ± 0.041	1.05 ± 0.03	620.8 ± 39.9 ^a^
2% HPMC K15M	24.08 ± 2.13 ^b^	223.43 ± 20.02 ^b^	0.940 ± 0.181	1.11 ± 0.05	3028.2 ± 270.2 ^b^
2% HPMC K100M	13.30 ± 0.42 ^c^	122.89 ± 3.87 ^c^	0.797 ± 0.180	1.03 ± 0.08	1768.0 ± 230.6 ^c^
2% PCP:HPMC K15M	7.53 ± 0.82 ^d^	70.16 ± 7.83 ^d^	0.365 ± 0.162	1.08 ± 0.07	913.2 ± 111.1 ^a^
3% PCP:HPMC K15M	6.14 ± 0.70 ^a^	57.09 ± 6.55 ^a^	0.272 ± 0.027	1.09 ± 0.04	808.4 ± 63.8 ^a^
3% PCP:HPMC K100M	5.25 ± 0.89 ^a^	49.10 ± 8.31 ^a^	0.294 ± 0.037	1.06 ± 0.02	713.1 ± 89.7 ^a^

Mean ± SD, *n* = 4. ^a–d^ Within columns, means with a different superscript letter are significantly different (*p* < 0.05, one-way ANOVA followed by Tukey’s post hoc multiple comparisons). *K*_0_ and *K_H_* are zero-order and Higuchi release rates (6–48 h), respectively. *K_KP_* refers to the Korsmeyer–Peppas rate constant calculated during the time range that the fractional DA released was ≤60% (%/h*^n^*), while *n* refers to the release exponent parameter. *Q*_72h_ refers to the cumulative released in μg at 72 h.

**Table 5:** Physical and chemical stability of selected *C. comosa* mucoadhesive gels after storage at 5 ± 3 °C (long-term) and 30 ± 2 °C/75 ± 5% RH (accelerated) conditions after 6 and 12 months, respectively, compared with fresh gels.

Formulations	Physical Properties		Percentage Remaining of DAs	
	pH	Percentage Viscosity (%)	DA1	DA2
2% PCP				
Fresh gels	4.63 ± 0.01 ^a^	100.00 ± 2.38 ^a^	100.00 ± 1.38 ^a^	100.00 ± 2.01 ^a^
6 mo accelerated-aged gels	4.78 ± 0.01 ^c^	97.63 ± 3.37 ^a^	42.06 ± 0.34 ^b^	103.95 ± 1.05 ^a^
6 mo long-term-aged gels	4.66 ± 0.02 ^a^	98.35 ± 6.67 ^a^	99.12 ± 1.99 ^a^	103.52 ± 2.44 ^a^
12 mo long-term-aged gels	4.71 ± 0.01 ^b^	89.99 ± 4.28 ^a^	98.97 ± 4.46 ^a^	103.89 ± 2.78 ^a^
3% PCP				
Fresh gels	4.56 ± 0.02 ^a^	100.00 ± 4.42 ^a^	100.00 ± 2.67 ^a^	100.00 ± 2.79 ^a^
6 mo accelerated-aged gels	4.72 ± 0.01 ^c^	98.84 ± 2.81 ^a^	45.74 ± 1.13 ^b^	102.82 ± 3.10 ^a^
6 mo long-term-aged gels	4.59 ± 0.01 ^a^	99.09 ± 2.05 ^a^	99.07 ± 1.36 ^a^	103.37 ± 1.85 ^a^
12 mo long-term-aged gels	4.68 ± 0.01 ^b^	90.95 ± 4.68 ^a^	95.46 ± 3.52 ^a^	103.58 ± 4.09 ^a^
2% PCP:HPMC K15M				
Fresh gels	4.64 ± 0.02 ^a^	100.00 ± 2.08 ^a^	100.00 ± 1.36 ^a^	100.00 ± 1.59 ^a^
6 mo accelerated-aged gels	4.84 ± 0.01 ^b^	94.42 ± 2.97 ^a^	61.06 ± 1.70 ^b^	102.11 ± 1.53 ^a^
6 mo long-term-aged gels	4.66 ± 0.01 ^a^	99.95 ± 4.66 ^a^	100.40 ± 2.89 ^a^	102.55 ± 3.97 ^a^
12 mo long-term-aged gels	4.81 ± 0.01 ^b^	93.46 ± 6.05 ^a^	100.12 ± 3.34 ^a^	103.54 ± 4.17 ^a^
3% PCP:HPMC K15M				
Fresh gels	4.56 ± 0.01 ^a^	100.00 ± 3.77 ^a^	100.00 ± 1.86 ^a^	100.00 ± 1.79 ^a^
6 mo accelerated-aged gels	4.83 ± 0.01 ^b^	93.47 ± 2.60 ^a^	66.22 ± 2.04 ^b^	101.80 ± 2.00 ^a^
6 mo long-term-aged gels	4.60 ± 0.02 ^a^	99.77 ± 2.34 ^a^	100.59 ± 3.06 ^a^	101.19 ± 3.87 ^a^
12 mo long-term-aged gels	4.81 ± 0.02 ^a^	92.75 ± 4.52 ^a^	99.21 ± 3.76 ^a^	102.48 ± 3.74 ^a^

Mean ± SD, *n* = 3. ^a–c^ Within columns, means with a different superscript letter are significantly different (*p* < 0.05, one-way ANOVA followed by Tukey’s post hoc multiple comparisons). Percentage viscosity refers to values of viscosity relative to the value of fresh gels.

## Data Availability

Data available on request.

## Supplementary Materials

The following supporting information can be downloaded at: https://www.mdpi.com/article/10.3390/pharmaceutics15010264/s1, Figure S1: The force–time curve (a), force–distance curve (b), and detachment force–time curve (c) indicated the mechanical properties, extrudability, and mucoadhesiveness of PCP-based C. comosa gels determined from TA-XTplus texture analyzer. Figure S2: Schematic illustration of mucoadhesive test via texture analyzer coupled with 10 mm diameter cylinder probe and porcine vaginal mucosa at 25 ± 1 °C. Figure S3: Macroscopic characteristics of placebo gels (gels without *C. comosa* extract) prepared from various mucoadhesive polymers.
