# Supplementary material for: Formulation and In Vitro Evaluation of Mucoadhesive Sustained Release Gels of Phytoestrogen Diarylheptanoids from Curcuma comosa for Vaginal Delivery
Source: Pharmaceutics. 2023 Jan 12;15(1):264. doi: 10.3390/pharmaceutics15010264 (PMC9862155; doi:10.3390/pharmaceutics15010264)
Supplement: Supplementary file 1 [file pharmaceutics-15-00264-s001.zip › pharmaceutics-2120427-supplementary.pdf]

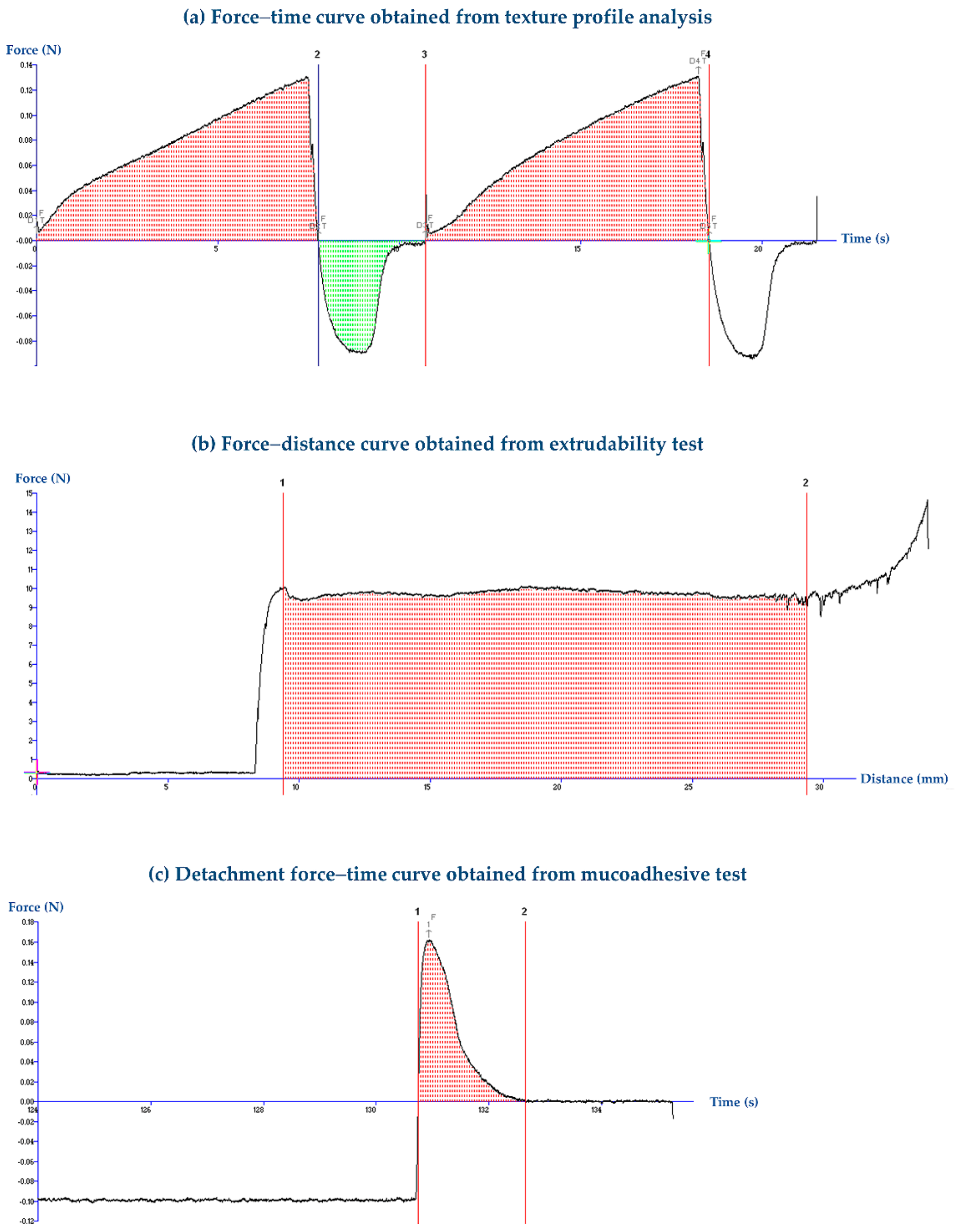

Figure S1: The force–time curve (a), force–distance curve (b), and detachment force–time curve (c) indicated the mechanical properties, extrudability, and mucoadhesiveness of PCP-based *C. comosa* gels determined from TA-XTplus texture analyzer.

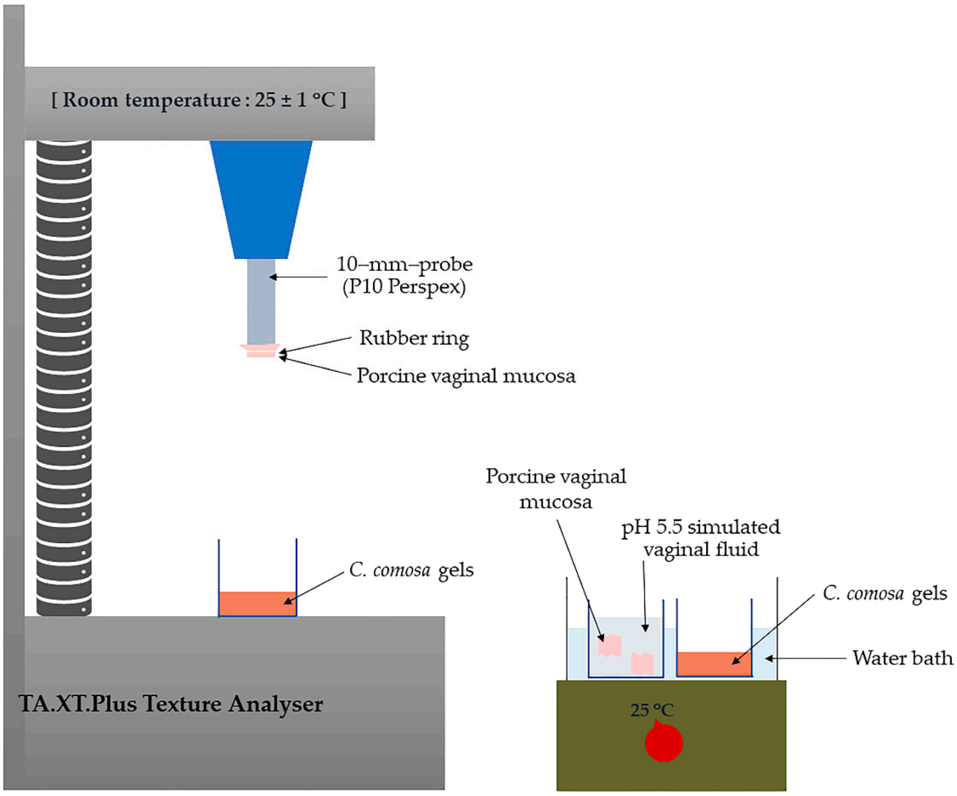

Figure S2: Schematic illustration of mucoadhesive test via texture analyzer coupled with 10 mm diameter cylinder probe and porcine vaginal mucosa at 25 ± 1 °C.

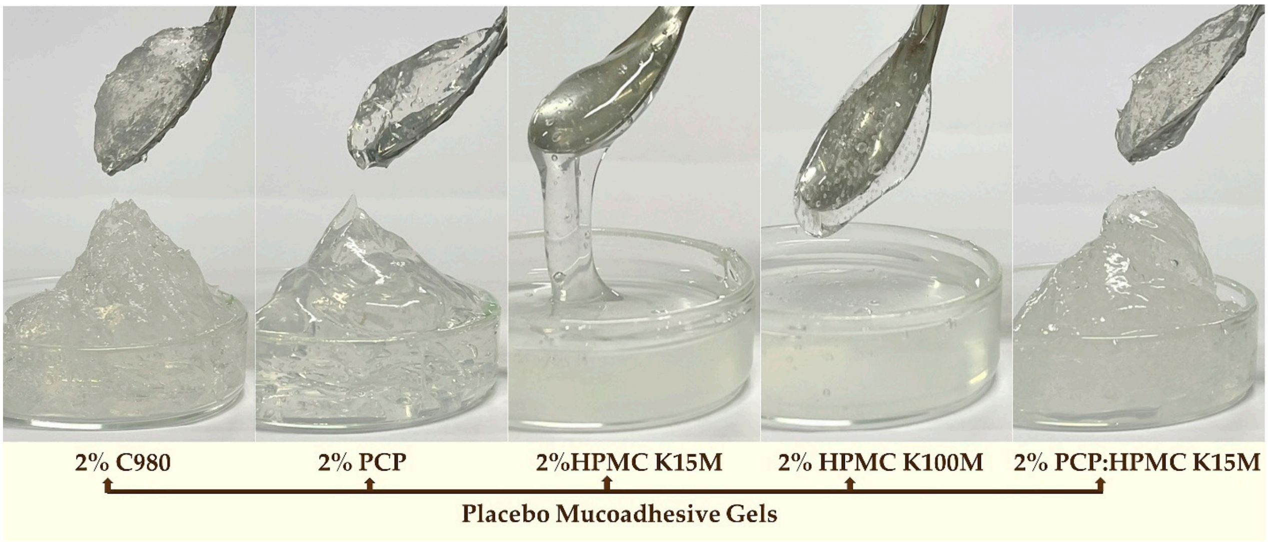

Figure S3: Macroscopic characteristics of placebo gels (gels without *C. comosa* extract) prepared from various mucoadhesive polymers.
